# Safe Discharge Home With Telemedicine of Patients Requiring Nasal Oxygen Therapy After COVID-19

**DOI:** 10.3389/fmed.2021.703017

**Published:** 2021-11-03

**Authors:** Aurélien Dinh, Jean-Christophe Mercier, Luc Jaulmes, Jean-Yves Artigou, Yves Juillière, Youri Yordanov, Patrick Jourdain, The AP-HP/Universities/INSERM COVID-19 Research Collaboration, Dinh Aurélien

**Affiliations:** ^1^Infectious Disease Department, University Hospital Raymond-Poincaré, Assistance Publique–Hôpitaux de Paris, Paris Saclay University, Garches, France; ^2^COVIDOM, Assistance Publique–Hôpitaux de Paris, Paris, France; ^3^Centre de Pharmaco-épidémiologie (Cephepi), Pitié Salpêtrière Hospital, Paris, France; ^4^Emergency Department, University Hospital Saint-Antoine, Assistance Publique–Hôpitaux de Paris, Sorbonne University, Paris, France; ^5^Cardiology Department, University Hospital Bicêtre, Assistance Publique–Hôpitaux de Paris, Paris Saclay University, Le Kremlin-Bicêtre, France

**Keywords:** COVID-19, pneumonia, oxygen therapy, home monitoring, telesurveillance

## Abstract

**Introduction:** The COVID-19 pandemic created challenges to healthcare systems worldwide. To allow overwhelmed hospitals to focus on the most fragile and severely ill patients, new types of management had to be set up. During the pandemic, patients with COVID-19 from greater Paris area were monitored at home using a web-based remote system called COVIDOM™, using self-administered questionnaires, which triggered alerts to a regional control center. To ease hospital discharge and to prevent hospital from being overwhelmed, patients still requiring low-flow oxygen therapy discharged home were also included in this telemedicine solution. We aim to evaluate the safety of this original management.

**Methods:** We conducted a retrospective multicenter cohort of patients discharged home from hospital after COVID-19 and still requiring nasal oxygen therapy, who were monitored by questionnaire and trained physicians using COVIDOM. During late follow-up, the status of the patients using a Euro-Qol (EQ-5D-5L) questionnaire, and the Medical Research Council (MRC) Dyspnea scale was collected.

**Results:** From March 21st to June 21st 2020, 73 COVID-19 patients still receiving nasal oxygen at hospital discharge were included. Median [Inter-Quartile Range (IQR)] age was 62.0 [52.5–69.0] years, 64.4% were male. Altogether, risk factors were observed in 49/73 (67%) patients, mainly hypertension (35.6%), diabetes mellitus (15.1%) and active neoplasia (11.0%). Among the cohort, 26% of patients were previously managed in ICU. Oxygen therapy was required for a median [IQR] of 20 [16–31] days. No death or urgent unplanned hospitalization were observed during the COVIDOM telemonitoring. During the late follow-up evaluation (6 months after inclusion), the mean EQ-5D-5L questionnaire score was 7.0 ± 1.6, and the mean MRC dyspnea scale was 0.8 ± 1.0, indicating absence of dyspnea. Five patients have died from non-COVID causes.

**Conclusions:** In this preliminary study, early discharge home of patients with severe COVID-19 disease who still required low-oxygen therapy seems to be safe.

## Introduction

During the COVID-19 pandemic, as in many countries, the French health system was overwhelmed by the first wave in early Spring 2020, with saturation of hospital beds. To help hospitals manage patients with COVID-19, several telemedicine-enabled early discharge of patients hospitalized with COVID-19 were set up worldwide (1–3).

A telesurveillance solution named COVIDOM™ was also deployed in the greater Paris area to monitor patients with COVID-19 at home, as part of an outpatient care or at hospitalization discharge (4). However, after acute respiratory tract infections, patients requiring oxygen therapy are usually discharged home once they are weaned from oxygen, despite other biological parameters being back to normal (5). Thus, home oxygen monitoring could provide substantial bed savings in acute care during the pandemic.

Therefore, we set up a cohort of COVID-19 patients discharged home under nasal oxygen therapy with an electric-powered oxygen extractor and a pulse oximeter, and monitored by COVIDOM™ (4). We aim to evaluate the safety of this original management.

## Methods

We retrospectively included all patients discharged home from hospital after COVID-19 requiring nasal oxygen therapy ( ≤ 4L/min), who were monitored using COVIDOM™, a web application used during the initial outpatient management or at hospital discharge after a COVID-19 related hospitalization. Patients with a suspected or microbiological confirmed case of COVID-19 were registered in COVIDOM™ by a physician after receiving a brief information and giving oral consent. At registration, patients would fill in a medical questionnaire on comorbidities, risk factors and symptoms. They would subsequently receive daily monitoring questionnaires for the duration of follow-up (until oxygen therapy withdrawal) (presented in Appendix 1). In case of abnormal responses, alerts were triggered to a regional control center.

The number and types of alerts generated by the answers to daily questionnaires were recorded. A red alert suggested possible deterioration of the patient's condition. In case of answers above a certain threshold, an orange alert was emitted. Finally, a gray alert would mean missing data. Depending on the types of alerts (top priority, mild priority, no answer, respectively), the patient was called back to check his/her status and would either received medical advice, was referred to his/her practitioner, or was hospitalized through the medical emergency transportation system.

Moreover, these patients received daily phone counseling on the management of oxygen therapy by an expert physician. Nasal oxygen flow was set up to maintain pulsed oxygen saturation (SpO2) within the physiologic range of 93–97%. Careful attention was paid to the respiratory rate and SpO2, both at rest and during daily domestic activities (e.g., toilet, shower, etc.).

In this study, clinical and biological data were collected from the COVIDOM database and the French Health Data Hub. During a late follow-up (at least 6 months after hospital discharge), a short survey was performed by phone by the same investigator (JCM) about the status of the patients using a Euro-Qol (EQ-5D-5L) questionnaire (6), and the Medical Research Council (MRC) Dyspnea scale (7) (detailed in Appendix 1).

The characteristics of patients were presented with frequencies and percentages for qualitative variables and mean (standard deviation) or median (interquartile [IQR]) as appropriate for quantitative variables.

To identify factors associated with longer duration of nasal oxygen therapy, a univariate analysis by logistic regression was performed, using demographic and medical characteristics, as well as all clinical and biological data from the COVIDOM database. Longer duration was defined as superior or equal to the median duration of oxygen therapy found in the study population.

Analyses were performed with the use of R software, version 3.6.1 (R Foundation for Statistical Computing).

All patients provided written informed consent. This study was approved by the scientific and ethical committee of APHP (IRB00011591).

## Results

From March 21st to June 21st 2020, 73 COVID-19 patients still receiving nasal oxygen at hospital discharge were managed through the COVIDOM solution (Table 1). Median [IQR] age was 62.0 [52.5–69.0] years, 64.4% were male patients. Altogether, risk factors were observed in 49/73 (67%) patients. The three main risk factors were hypertension (35.6%), diabetes mellitus (15.1%) and neoplasia (11.0%). Among the cohort, 26% of patients were managed in intensive care unit (ICU) during their hospitalization. Median [IQR] length of stay in ICU was 8.7 [5.7–13.3] days and the median [IQR] length of stay in hospital was 18.8 [10.9–26.0] days.

**Table 1:** Characteristics of the COVID-19 patients receiving nasal low-flow oxygen at hospital discharge, as compared with those not receiving oxygen.

	**Patients**
Number of patients	73
Male patients (*n*, %)	47 (64.4)
Median age [IQR] (years)	62.0 [52.5–69.0]
Suspected COVID-19 or confirmed by PCR	62/11
Median [IQR] days between first symptoms and registry into COVIDOM™	19.2 [14.6–27.5]
Median [IQR] days of hospitalization	18.8 [10.9–26.0]
Median [IQR] days spent in the ICU	8.7 [5.7–13.3]
Presence of risk factors (*n*, %)	49/24
Obesity	4 (5.5)
Hypertension	26 (35.6)
Congestive heart failure	3 (4.1)
Diabetes	11 (15.1)
Asthma	6 (8.2)
Chronic obstructive pulmonary disease	3 (4.1)
Immunosuppression, transplant	3 (4.1)
Cancer, chemotherapy	8 (11.0)
Cirrhosis	3 (4.1)
**Ventilation during hospitalization (n, %)**	
Non-invasive mechanical ventilation	14 (19.2)
Invasive mechanical ventilation	5 (6.8)
**Etiologic therapy (** ***n*** **, %)**	
Lopinavir-retronavir	3 (4.1)
Chloroquine and/or azithromycin	28 (38.4)
Steroids	12 (16.4)
Median [IQR] days of oxygen therapy	20.0 [8.0–28.0]
**Late follow-up (** ***n*** **, %)**	
Alive/dead	68/5
Re-hospitalization	0
EuroQOL questionnaire score (mean ± SD)^*^	7.0 ± 1.6
MRC dyspnea scale (mean ± SD)^†^	0.8 ± 1.0
Median [IQR] delay of follow-up (weeks)	41.1 [39.7-41.8]

* *Excellent health status is 6 (1.1.1.1.1.1.) – intermediate health status between 9–11 – poor health status is 15 (3.3.2.2.3.2)*.

† *Medical Research Council dyspnea grading: 0: no dyspnea; 1: exertional dyspnea; 2: mild effort dyspnea leading to stop walking; 3: marked effort dyspnea limiting walking; 4: dyspnea at rest or keeping home. ICU, intensive care unit; IQR, interquartile range; MRC, Medical Research Council; SD, standard deviation*.

**Figure 1:**
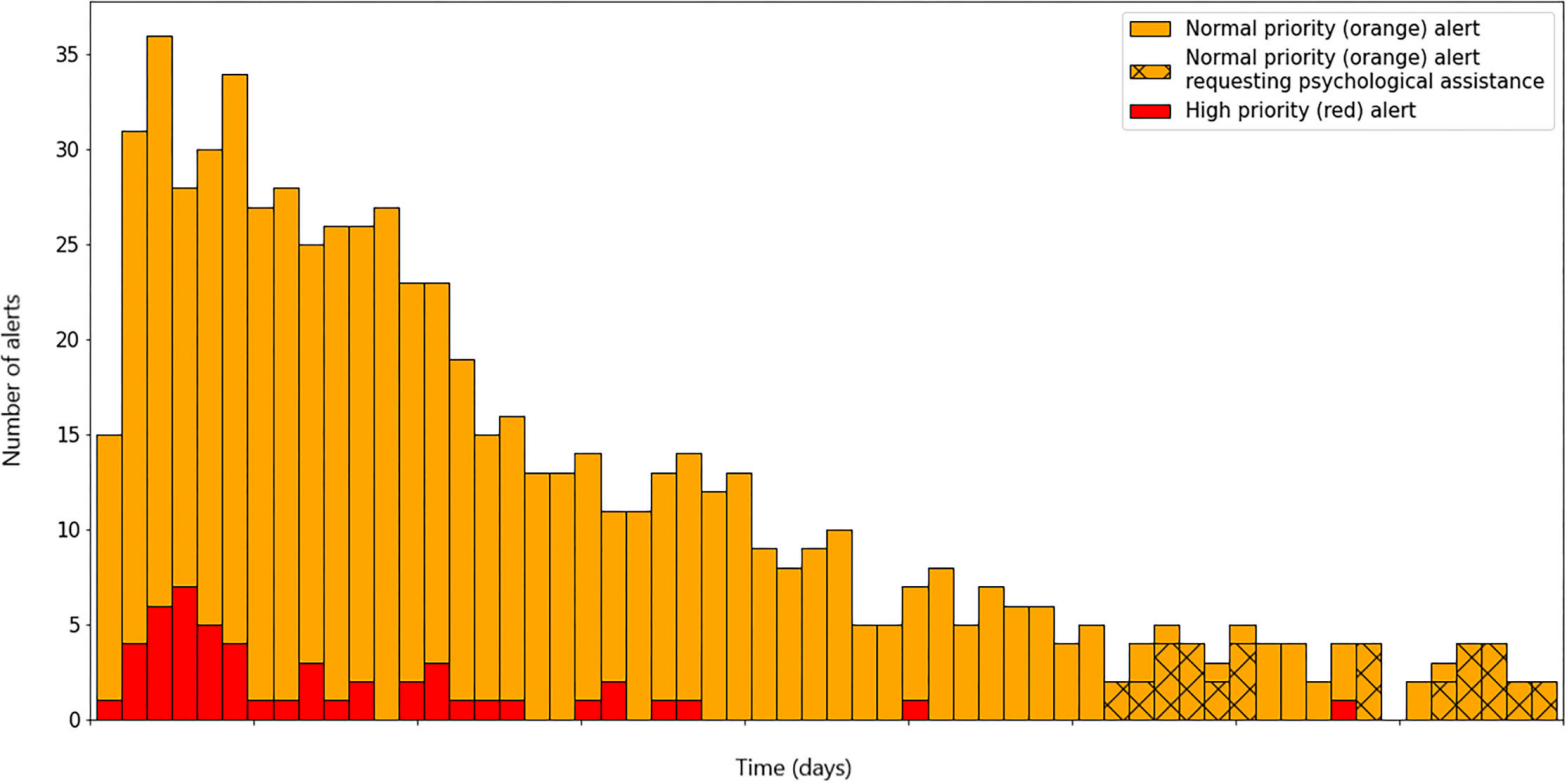
Bar plot of alert on COVIDOM platform generated by patient with nasal oxygen.

Overall, 738 red or orange alerts from 38 patients were generated (Figure 1). Main causes were respiratory issues: high respiratory rate (73.7%), mild dyspnea (52.8%), low oxygen saturation below 94% (28.6%).

Oxygen therapy of 4L/min or less was required for a median [IQR] of 20 [16–31] days. Altogether, the 1,337 days of oxygen therapy at home allowed to save about 70 hospital beds for 20 days.

No death and no urgent unplanned hospitalization were observed during the COVIDOM telemonitoring. The late follow-up evaluation was performed after a median [IQR] delay of 41.1 [39.7–41.8] weeks after hospital discharge.

The mean EQ-5D-5L questionnaire score was 7.0 ± 1.6, suggesting patients were in good health (<8/15). Mean MRC dyspnea scale was 0.8 ± 1.0, indicating absence of dyspnea. Five patients died during follow-up: 4 from underlying diseases, i.e. pancreatic cancer, bone marrow transplant complicated by JCV virus meningitis, glioblastoma, and general state alteration, and one from a car crash accident.

Furthermore, in the univariate analysis, when comparing patients with a longer duration of nasal oxygen therapy (≥ 20.0 days) (Table 2), the only associated factor was hypertension.

**Table 2:** Univariate analysis of variables associated with longer duration of nasal oxygen therapy (n = 65).

	**Patients with prolonged oxygen therapy (≥20 Days) N = 24**	**Patients with shorter oxygen therapy (<20 days)N = 41**	**P-value**
Male gender	16 (66.7)	26 (63.4)	0.7909
Age (median [IQR], years)	66.0 [54.8; 74.3]	61.0 [51.0; 68.0]	0.0933
**Risk factor**			
Obesity	1 (4.2)	3 (7.3)	0.6506
Hypertension	14 (58.3)	10 (24.4)	**0.0070**
Congestive heart failure	1 (4.2)	2 (4.9)	0.8589
Diabetes melitus	5 (20.8)	5 (12.2)	0.4298
Chronic obstructive pulmonary disease, asthma	4 (16.7)	4 (9.8)	0.4536
Active neoplasia	2 (8.3)	6 (14.6)	0.4176
Managed in ICU during hospitalization	6 (25.0)	9 (22.0)	0.7702
Invasive ventilation during hospitalization	2 (8.3)	3 (7.3)	1.0000

*Data are n (%) unless otherwise stated. ICU, intensive care unit; IQR, interquartile range. Bold values means statistically significant (as inferior to 0.05)*.

## Discussion

This experience demonstrated the ability to safely discharge patients requiring low-flow nasal oxygen therapy after COVID-19. It is of note that patients were carefully monitored daily by telemedicine and expert physicians. This management might have contributed to relieve a hard pressure on desperately needed beds.

The only factor associated with longer oxygen nasal therapy was hypertension, which has been already described as a risk factor for severe COVID-19 disease (8). The small sample size could possibly lead to non-significant result for other factors.

Nevertheless, we present only a small cohort with preliminary results with no control group.

In Italy, an experience of telemedicine was set up to discharge mild COVID-19 patients to repurposed hotel rooms, with nurses and physicians (1). Its purpose was to accelerate discharge of low-dependency patients into isolation, pending PCR-swab results, because these mildly severe patients could not be discharged to their homes for fear of infecting their household. Therefore, hotels were playing the role of auxiliary hospitals, for COVID-19 patients requiring ongoing monitoring of vital signs before discharge. Overall, 258 patients with COVID-19 were discharged from the hospital to the hotel within two months, at the peak of the pandemic in Roma.

Another model named “COVID-19 Intermediate Care” was developed in Canada, in the Saskatchewan province (2). This model involved primary care physicians. Indeed, patients had to input their own vital signs (i.e. temperature, heart rate, blood pressure and oxygen saturation) as well as answer to a COVID-19 digital questionnaire twice daily, via specific tools and digital media. In case of abnormalities in their clinical data, yellow and red flags were prompted, and the physician could then interact with the patients through video-consultation or in-person visit, if necessary. Within a month, a total of 962 patients were enrolled, which prevented the Saskatchewan hospitals from being overwhelmed.

In contrast with these other international experiences that aimed to triage patients with COVID-19 toward the most appropriate facility, ours targeted post-hospitalization care with often severe diseases (9, 10).

Our results are in line with a recent cohort study performed in the United States which showed that patients discharged on home oxygen had low rates of mortality and rehospitalization (11). This study has several limitations. Firstly, it is an observational study with potential bias considering for indication and selection of patients. Moreover, no control group is available.

We believe that our original experience deserves to be shared with other countries, particularly in the context of the possible next wave of the pandemic currently encountered in Europe and elsewhere.

## Data Availability Statement

The raw data supporting the conclusions of this article will be made available by the authors, without undue reservation.

## Ethics Statement

The studies involving human participants were reviewed and approved by Assistance Publique - Hopitaux de Paris (APHP). The patients/participants provided their written informed consent to participate in this study.

## AP-HP/Universities/Inserm COVID-19 Research Collaboration Members

Writing committee: Dinh Aurélien (Infectious Disease Department, University Hospital Raymond-Poincaré, Assistance Publique – Hôpitaux de Paris, Garches, Paris Saclay University, Garches, France); Mercier Jean-Christophe, Artigou Jean-Yves, Juillière Yves (COVIDOM, Assistance Publique – Hôpitaux de Paris, Paris, France); Jaulmes Luc (Centre de Pharmaco-Épidémiologie (Cephepi), Pitié Salpêtrière Hospital, Paris, France); Yordanov Youri (Emergency Department, University Hospital Saint-Antoine, Assistance Publique – Hôpitaux de Paris, Sorbonne University, Paris, France); Jourdain Patrick (Cardiology Department, University Hospital Bicêtre, Assistance Publique – Hôpitaux de Paris, Paris Saclay University, Le Kremlin-Bicêtre, France); Data-sciences committee: Apra Caroline (Sorbonne Université, AP-HP, Hôpital Pitié Salpêtrière, Service de Neurochirurgie, Paris, France); Jaulmes Luc (Centre de Pharmaco-Épidémiologie (Cephepi), Pitié Salpêtrière Hospital, Paris, France); Mensch Arthur (Ecole Normale Supérieure, PSL University, CNRS, Départment de Mathématiques et Applications, Paris, France); Scientific committee: Aime-Eusebi Amélie, Apra Caroline, Bleibtreu Alexandre, Debuc Erwan, Dechartres Agnes, Deconinck Laurene, Dinh Aurelien, Jourdain Patrick, Katlama Christine, Lebel Josselin, Lescure François-Xavier, Yordanov Youri; Covidom regional center steering commitee: Artigou Yves, Banzet Amelie, Boucheron Elodie, Boudier Christiane, Buzenac Edouard, Chapron Marie-Claire, Chekaoui Dalhia, De Bastard Laurent, Debuc Erwan, Dinh Aurelien, Grenier Alexandre, Haas Pierre-Etienne, Hody Julien, Jarraya Michele, Jourdain Patrick, Lacaille Louis, Le Guern Aurelie, Leclert Jeremy, Male Fanny, Marchand-Arvier Jerome, Martin-Blondet Emmanuel, Nassour Apolinne, Ourahou Oussama, Penn Thomas, Ribardiere Ambre, Robin Nicolas, Rouge Camille, Schmidt Nicolas, Villie Pascaline.

## Author Contributions

AD and J-CM conceived and designed the study. All authors collected, interpreted the data, and critically reviewed the manuscript. LJ performed the statistical analysis. AD and J-CM drafted the manuscript. All authors contributed to the article and approved the submitted version.

## Conflict of Interest

The authors declare that the research was conducted in the absence of any commercial or financial relationships that could be construed as a potential conflict of interest.

## Publisher's Note

All claims expressed in this article are solely those of the authors and do not necessarily represent those of their affiliated organizations, or those of the publisher, the editors and the reviewers. Any product that may be evaluated in this article, or claim that may be made by its manufacturer, is not guaranteed or endorsed by the publisher.
