# Supplementary material for: Safe Discharge Home With Telemedicine of Patients Requiring Nasal Oxygen Therapy After COVID-19
Source: Front Med (Lausanne). 2021 Nov 3;8:703017. doi: 10.3389/fmed.2021.703017 (PMC8595095; doi:10.3389/fmed.2021.703017)
Supplement: Supplementary file 1 [file Table_1.DOCX]

**Appendix 1: COVIDOM-O2 questionnaire**

**INCLUSION**

**Clinical signs:**

During your infection, which clinical signs did you present?

Fever (temperature >38°C)

Chills

Cough

Dyspnea

Fatigue

Muscular aches

Nausea, vomiting

Loss of taste

Loss of appetite

Loss of smell

Thoracic oppression during several minutes

Thoracic pain

Diarrhea

Chilblains

Cutaneous eruption, rash

Conjunctivitis

Other signs

**Treatments**

Which treatment did you receive for COVID?

Aspirin

Azithromycine

Hydrocholoroquine

Rovamycin

Corticoids (prednisolone/cortancyl/celestene)

Paracetamol

Nonsteroidal anti-inflammatory drugs

Remdesivir

Lopinavir/ritonavir

Other

None

**Risk factors:**

Do you suffer from:

Cancer (under ongoing treatment)

Diabetes

Renal failure

Heart attack or angina pectoris

Heart failure

High blood pressure

Asthma

COPD or emphysema

Other chronic disease, if so specify

Do you smoke?

If yes, for how many years? How many a day?

Did you stop smoking since the onset of COVID-19?

**FOLLOW-UP:**

**Clinical signs:**

On this day, which clinical signs are you presenting?

Fever (temperature >38°C)

Chills

Cough

Dyspnea

Fatigue

Muscular aches

Nausea, vomiting

Loss of taste

Loss of appetite

Loss of smell

Thoracic oppression during several minutes

Thoracic pain

Diarrhea

Chilblains

Cutaneous eruption, rash

Conjunctivitis

Other signs

**Treatments**

Which treatment did you receive for COVID since the last contact?

Aspirin

Azithromycine

Hydrocholoroquine

Rovamycin

Corticoids (prednisolone/cortancyl/celestene)

Paracetamol

Nonsteroidal anti-inflammatory drugs

Remdesivir

Lopinavir/ritonavir

Other

None

**Intercurrent events**

Since the last contact,

Did you contact the emergency services?

Did you go to the Emergency Room?

Did you see your physician during a home visit?

Did you see your physician during a consultation?

Did you see your physician during a teleconsultation?

Were you hospitalized? If so specify which hospital?

Were you given a CT-scan for your infection?

Were you given a chest X-ray for your infection?

Were you able to return to usual activities?

**LATE FOLLOW-UP:**

Since the last contact,

Were you hospitalized? If so specify which hospital?

**MRC dyspneoa scale:**

| Grade | Degree of breathlessness related to activity |
| --- | --- |
| 0 | Not troubled by breathless except on strenuous exercise |
| 1 | Short of breath when hurrying on a level or when walking up a slight hill |
| 2 | Walks slower than most people on the level, stops after a mile or so, or stops after 15 minutes walking at own pace |
| 3 | Stops for breath after walking 100 yards, or after a few minutes on level ground |
| 4 | Too breathless to leave the house, or breathless when dressing/undressing |

**EuroQol questionnaire:**

**Mobility**

1. I have no problems in walking about

2. I have some problems in walking about

3. I am confined in bed

**Self-care**

1. I have no problems with self-care

2. I have some problems washing or dressing myself

3. I am unable to wash or dress myself

**Usual activities**

1. I have no problems with performing my usual activities

2. I have some problems with performing my usual activities

3. I am unable to perform my usual activities

**Pain/discomfort**

1. I have no pain or discomfort

2. I have moderate pain or discomfort

3. I have extreme pain or discomfort

**Anxiety/depression**

1. I am not anxious or depressed

2. I am moderately anxious or depressed

3. I am extremely anxious or depressed
